# Effects of Sesame (*Sesamum indicum* L.) Supplementation on Creatine Kinase, Lactate Dehydrogenase, Oxidative Stress Markers, and Aerobic Capacity in Semi-Professional Soccer Players

**DOI:** 10.3389/fphys.2017.00196

**Published:** 2017-03-31

**Authors:** Carlos V. da Silva Barbosa, Alexandre S. Silva, Caio V. C. de Oliveira, Nayara M. L. Massa, Yasmim R. F. de Sousa, Whyara K. A. da Costa, Ayice C. Silva, Plínio Delatorre, Rhayane Carvalho, Valdir de Andrade Braga, Marciane Magnani

**Affiliations:** ^1^Laboratory for Physical Training Studies Applied to Performance and Health, Federal University of ParaíbaJoão Pessoa, Brazil; ^2^Laboratory of Biochemistry of Foods, Department of Food Engineering, Federal University of ParaíbaJoão Pessoa, Brazil; ^3^Embrapa CottonCampina Grande, Brazil; ^4^Department of Molecular Biology, Federal University of ParaíbaJoão Pessoa, Brazil; ^5^Biotechnology Center, Federal University of ParaibaJoao Pessoa, Brazil

**Keywords:** athletes, oxidative stress, muscle damage, inflammation, functional food

## Abstract

Nutritional intervention with antioxidants rich foods has been considered a strategy to minimize the effects of overtraining in athletes. This experimental, randomized, and placebo-controlled study evaluated the effects of consumption of sesame (*Sesamum indicum* L.) on muscle damage markers, oxidative stress, systemic inflammation, and aerobic performance in male semi-professional soccer players. Twenty athletes were randomly assigned to groups that received 40 g (two tablespoons) per day of sesame or a placebo during 28 days of regular training (exposed to routine training that includes loads of heavy training in the final half of the season). Before and after intervention, creatine kinase (CK), lactate dehydrogenase (LDH), malondialdehyde (MDA), superoxide dismutase (SOD), C-reactive protein (hs-CRP), and aerobic capacity were evaluated. Before intervention, a physiologic imbalance was noted in both groups related to CK and LDH levels. Sesame intake caused a reduction of CK (19%, *p* < 0.05), LDH (37%, *p* < 0.05), MDA (55%, *p* < 0.05) and hs-CRP (53%, *p* < 0.05) and increased SOD (14%, *p* < 0.05), vitamin A (25%, *p* < 0.05), and vitamin E (65%, *p* < 0.05) in the experimental group. These phenomena were accompanied by increased aerobic capacity (17%, *p* < 0.05). The placebo group showed an increase in CK (5%, *p* < 0.05) and no significant change in LDH, SOD or vitamin A. MDA levels decreased (21%, *p* < 0.05) and vitamin E increased (14%, *p* < 0.05) in the placebo group, but to a much lesser extent than in the experimental group. These results show that sesame consumption may reduce muscle damage and oxidative stress while improving the aerobic capacity in soccer players.

## Introduction

To achieve maximum performance, athletes often face high-intensity, and/or high-volume physical training loads. Though this workout regime leads to high performance, it can also expose athletes to a non-functional risk of overreaching or overtraining. In such situations, an athlete's body cannot assimilate the training load and will show no improvement in performance or a sharp decline associated with neuro-immune endocrine disorders that affect athletes' health (Smith, 2004). In fact, it is estimated that even in young athletes, at least 20–30% have already experienced non-functional overreaching or signs of overtraining (Winsley and Matos, 2011).

Physical training naturally promotes damage of muscle tissues, which is accompanied by local inflammation. In the recovery period, muscle tissue repair and improvement of trained physical abilities occurs through the well-known phenomenon of general adaptation syndrome, which comprises non-specific systemic reactions of the body to continuous exposure to stress (Goldstein and Kopin, 2007; Aptekmann and Cesar, 2010; Winsley and Matos, 2011). However, if training loads are of high intensity and/or volume or the recovery period is inadequate, there may be an evolution from local muscle inflammation to systemic inflammation and oxidative stress, which evolves into neuroendocrine and behavioral disorders (Miranda-Vilela et al., 2009). Moreover, previous studies have shown minimization of muscle fatigue, systemic inflammation (Miranda-Vilela et al., 2009), and oxidative stress (Toscano et al., 2015) in athletes who received nutritional intervention with orange juice, pequi-fruit pulp oil, or grape juice, foods known for their antioxidant properties.

Sesame cultivar (*Sesamum indicum* L.) has high nutritional value due the significant amounts of dietary fiber, protein, natural antioxidants, unsaturated fatty acids, vitamins, and mineral constituents present in its composition (Anilakumar et al., 2010). The lipid fraction of sesame seeds shows good oxidative stability, which is attributed to the essential fatty acids, such as oleic, linoleic, and arachidonic present in its composition, as well as to antioxidant compounds (e.g., sesamin, sesamolin, and sesamol) that may act in synergism (Namiki, 2007; Kanu et al., 2010).

Previous study have demonstrated the antioxidant effects of sesame in hypertension model (Kamal-Eldin et al., 2011). Furthermore, the anti-inflammatory and antioxidant effects of sesame the anti-inflammatory and antioxidant effects of sesame via decreases in lipid peroxidation, C-reactive protein (hs-CRP), and interleukin-6 (IL-6) have also been shown (Haghighian et al., 2015). To date, whether soccer players would benefit from the antioxidant and anti-inflammatory effects of sesame has not been investigated. The aim of this study was to test the effect of a supplementation protocol with white sesame seeds (*S. indicum* L. cultivar BRS silk) on muscle damage markers, systemic inflammation, oxidative stress and aerobic capacity in soccer players exposed to routine training that includes loads of heavy training in the final half of the season.

## Methods

### Subjects

A group of 20 male semi-professional soccer players were randomly divided into an experimental (*n* = 10, age range 16–18 years) and a placebo group (*n* = 10, average range 16–18 years). The training routine consisted of weekly workouts of 6 days for 3 h per day. The exclusion criteria were articular problems, regular consumption of sesame, regular consumption of alcohol, smoking, lactose intolerance, use of medications for chronic diseases, and intake of minerals, vitamins, anabolic steroids, or similar compounds. The study was approved by the Committee on Ethical Research Involving Humans Beings of the Federal University of Paraíba (João Pessoa, Brazil) under the Protocol Number 04308612.2.0000.5183. All athletes had the permission of their legal guardian, who signed the informed consent as required by Resolution 196/96 of the Brazilian National Health Council (CNS).

### Experimental design

This was an experimental, randomized, and placebo-controlled study. After 10 months of training and games and 35 days before the last competition of the season, the athletes were submitted to the supplementation protocol with a sesame paste or placebo immediately before and after each training session for 28 days. Before and 48 h after the 28-day protocol, they performed tests for assessing aerobic capacity, and blood samples were collected to analyze the creatine kinase (CK), lactate dehydrogenase (LDH), high-sensitivity C-reactive protein (hs-CRP), malondialdehyde (MDA), superoxide dismutase (SOD), and vitamins A and E.

### Training history

In the 10 months prior to the start of the study, athletes participated in regular soccer training consisting of a brief pre-season aimed at the development of overall strength and aerobic endurance, followed by a regular season, during which the training focused on technical and tactical skills, strength, and speed. At the beginning of the study, athletes were preparing for a competition to be held 35 days after the beginning of the study. They had no official competitions during this period, only weekly friendly matches. During the supplementation period, all athletes maintained the regular season training schedule, which focused on the development of tactics, explosive strength and speed. The training consisted of one or two daily sessions. The athletes did not exercise in the 48 h preceding each blood collection and aerobic test to eliminate the acute effects of exercise.

### Preparation and administration of sesame paste and placebo

Seeds of the white sesame cultivar BRS silk (EMBRAPA-Brazil) were roasted at 105°C for 2 min and then crushed in a high-speed blender (Oster®) for 10 min. To facilitate consumption, 40 g of crushed seeds was mixed into a paste with 17 g of honey (*Apis mellifera*). The placebo sesame paste had similar physical characteristics as the sesame paste and had equal caloric content. It was made using 17 g of honey, maltodextrin, cow milk, and artificial caramel food coloring (Otkcer®, São Paulo, Brazil). The placebo and sesame pastes were placed in identical aluminum packages so that neither would be suspected of being the placebo. No differences between the texture and color of the placebo and sesame paste were detected in preliminary sensory studies (triangular test) performed with 60 students and professors at the Health Center of the Federal University of Paraíba (data not shown). The physicochemical composition according to the AOAC (AOAC, 2012) of the sesame paste was moisture 9.96/100 g, soluble fibers 19.35/100 g, insoluble fibers 1.88/100 g, ash 3.35/100 g; fat 51.58/100 g; and protein 20.88/100 g.

The athletes consumed 40 g (equivalent to two tablespoons) of sesame paste; this dose was based on a previous report on the benefits of sesame (Alipoor et al., 2012; Suwimol et al., 2012). The daily dose of sesame or placebo was consumed in two parts, one part before, and one part after one of the daily training sessions, except on game days, when the entire dose was administered just before game time. The researchers followed the training and managed the supplementation every day. Over the intervention period, the athletes were advised to maintain their dietary habits. A 24-h recall food survey was conducted weekly by trained interviewer (three times: 2 weekdays and 1 weekend day) to obtain information regarding food intake (Toscano et al., 2015). The average dietary intake was used to calculate the intake of nutrients using Avanutri Revolution software, version 4.0 (Avanutri, Rio de Janeiro, Brazil). Functional tests were performed to determine the running speed at the lactate threshold of 3.5 mM (V3.5 mM) and the peak velocity and peak oxygen consumption (peak VO2) following the Bruce protocol (Bruce et al., 1973) 48 h after the final training.

### Biochemical tests

After 12 h of fasting and before any exercise was performed that day, a blood sample (10 mL) was collected from the antecubital vein and centrifuged at 3,000 rpm for 15 min. The serum was collected and stored at −80°C for analysis of serum creatine kinase (CK), lactate dehydrogenase (LDH), malondialdehyde (MDA), and high-sensitivity C-reactive protein (hs-CRP). The CK and LDH enzymes were measured in the serum using the Labtest kit (Minas Gerais, Brazil) according to the manufacturer's instructions. The reading was performed in a Labmax Plenno at a wavelength of 340 nm. The SOD activity was determined by a RANSOD kit (Randox, USA) with readings of the red formazan performed with superoxide radical in a spectrophotometer at 505 nm following the manufacturer's instructions. MDA in the serum was determined using trichloroacetic acid, acid hydrolysis, and formation of a MDA-thiobarbituric acid complex. The MDA-(TBA)2 adduct was separated from other interfering compounds by C-18 reverse-phase HPLC techniques, with visible detection at 532 nm (Templar et al., 1999). The serum levels of vitamins A and E were measured using high-performance liquid chromatography (Dionex Ultimate 3000; Thermo Scientific, Mass., USA) at 325 nm for the quantification of vitamin A (retinol) and 295 nm for the quantification of vitamin E (α-tocopherol). The hs-CRP was assayed by immune turbidimetry using an automatic biochemical analyzer, Cobas Mira Plus (Roche), and the PCR Reagent Plus Ultra Turbiquest at 540 nm.

### Statistical analysis

The data are expressed as the mean and standard deviation of the mean. The independent *T*-test was performed to compare the initial values of the experimental and control groups. Two-way ANOVA (treatment by time) was performed to compare the differences between the treatments, considering *p* ≤ 0.05. All of the procedures were performed using GraphPad Prism 3.4 for Windows (GraphPad Software, San Diego, CA, USA).

## Results

Athletes in the experimental and placebo groups presented no differences regarding the assessed anthropometric variables (Table 1). The CK, LDH, MDA, and hs-CRP values obtained before the experimental intervention suggested physiological imbalances in the athletes included in the study, but no differences between groups were observed for any of these variables. SOD values were significantly lower in the experimental group than in the control group (Table 1).

**Table 1:** **Baseline characteristics of the groups before the intervention period**.

**Anthropometric variables**	**Experimental group (*n* = 10)**	**Placebo group (*n* = 10)**	*P*-value^*^
Weight (kg)	70.08 ± 4.52	68.02 ± 5.73	0.393
Height (cm)	179.60 ± 7.47	174.10 ± 6.04	0.103
Body mass index (kg/m^2^)	21.77 ± 1.51	22.45 ± 1.46	0.335
Body fat (%)	12.45 ± 2.14	13.76 ± 1.98	0.189
**FUNCTIONAL TESTS**			
Peak VO_2_ (ml.kg^−1^.min^−1^)	52.47 ± 4.10	52.61 ± 4.71	0.557
Peak velocity (km.h^−1^)	16.00 ± 1.33	16.05 ± 0.71	0.589
V_3.5*mM*_ (km.h^−1^)	12.02 ± 2.01	12.04 ± 2.33	0.716
**BIOCHEMICAL MARKERS**			
CK (U/L)	568.20 ± 32.18	515.90 ± 43.340	0.024
LDH (U/L)	537.40 ± 193.10	516.10 ± 231.40	0.850
MDA (μmol/L)	1.80 ± 1.00	1.90 ± 0.500	0.811
SOD (U/ml)	58 ± 1.50	60 ± 2.00	0.018
hs-CRP (mg/dL)	0.366 ± 0.14	0.331 ± 0.18	0.683
Vitamin A (μg/dL)	36.3 ± 3.1	37.2 ± 2.2	0.703
Vitamin E (μg/dL)	9.8 ± 1.8	10.5 ± 1.1	0.596

The weekly 24-h recalls showed that the two groups were similar with respect to nutritional intake (Table 2), and no differences were observed in the consumption of calories or of compounds recognized as antioxidants, such as vitamin C (±77 mg/day), vitamin E (±11 mg/day), or vitamin A (±790 mg/day). However, both groups demonstrated an initial deficiency in intake of vitamin A and vitamin E based on the recommendations of the DRI (Institute of Medicine, 2004).

**Table 2:** **Food intake of the athletes involved in the study**.

**Variables**	**Experimental group (*n* = 10)**	**Placebo group (*n* = 10)**	***P*-value**
Total calories (Kcal)	2470 ± 810.4	2993 ± 703.7	0.075
Carbohydrates (g/kg/day)	5.13 ± 1.6	6.53 ± 2.18	0.060
Carbohydrates (%)	58.44 ± 4.13	57.71 ± 4.36	0.643
Protein (g/kg/day)	1.39 ± 0.43	1.59 ± 0.39	0.198
Protein (%)	14.92 ± 2.69	14.78 ± 3.03	0.895
Lipids (g/kg/day)	1.06 ± 0.42	1.35 ± 0.36	0.057
Lipids (%)	26.64 ± 3.54	27.61 ± 3.68	0.471
Vitamin A (RE)	^*^789.8 ± 148.0	^*^698.4 ± 421.2	0.438
Vitamin C (mg)	677.5 ± 650.3	486 ± 415.3	0.434
Vitamin E (mg)	^*^9.73 ± 5.15	^*^10.95 ± 6.3	0.568

*Recommended daily dietary nutrient antioxidants by DRI's (2002/2005): Vitamin A: 900 μg; Vitamin C: 75 mg; Vitamin E: 15 mg*.

* *Indicates values below recommended. Dados are described as average ± standard deviation of the average*.

There were no reports of adverse symptoms such as bloating, cramping, nausea, vomiting, or diarrhea during the intervention, and no changes occurred in body mass index and body fat of the athletes, as assessed by skinfold measures.

**Figure 1:**
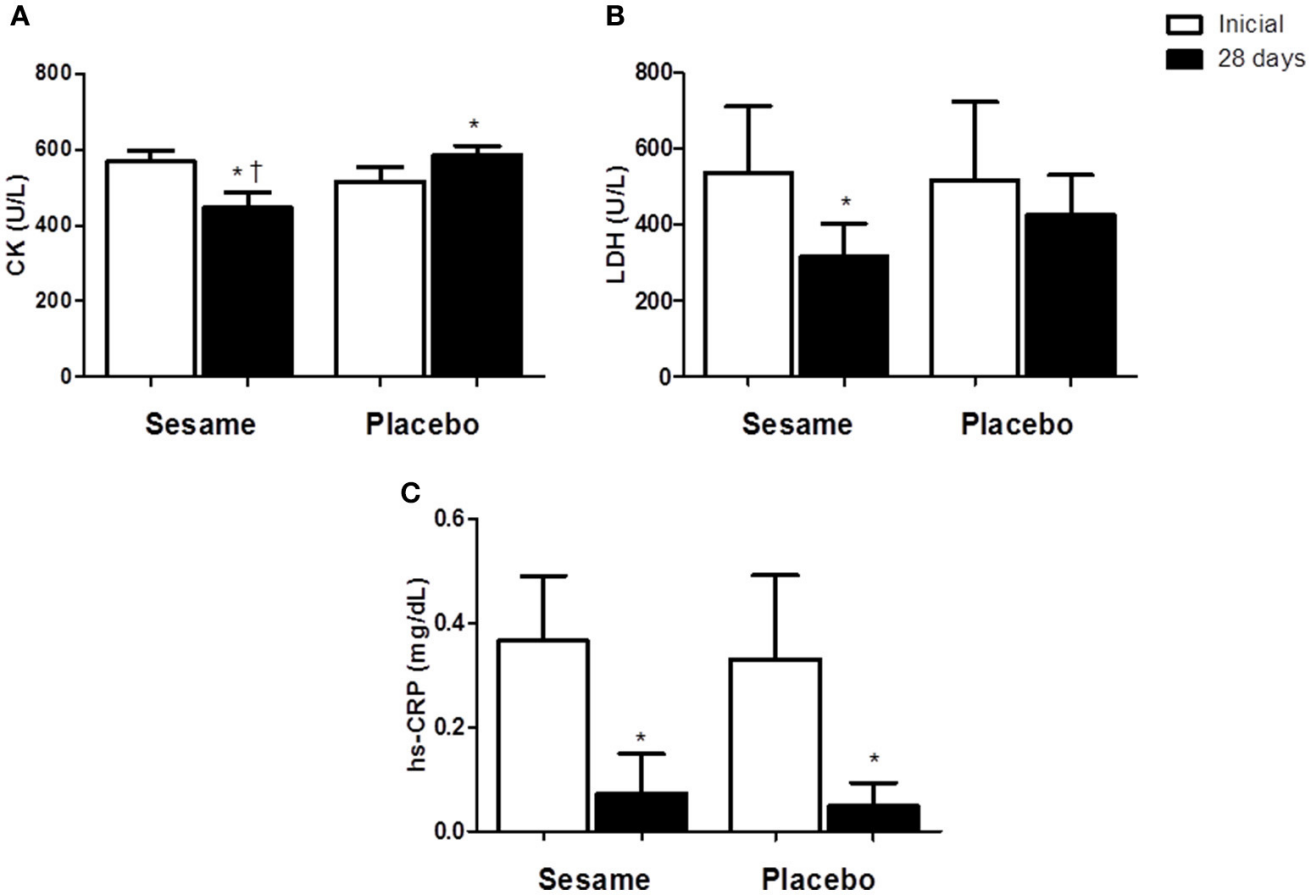
**Effect of sesame paste supplementation (***Sesamum indicum*** L.) on muscle damage markers (CK and LDH—A, B)** and systemic inflammation (hs-CRP, **C**). Data are expressed as average ± standard deviation. ^*^Indicates intra-group differences between pre- and post-supplementation; ^†^Indicates difference between groups at the end of the study (two-way ANOVA with Tukey's test). CK, creatinequinase enzyme; LDH, lactate dehydrogenase enzyme; hs-CRP, highly sensitive C reactivate protein.

**Figure 2:**
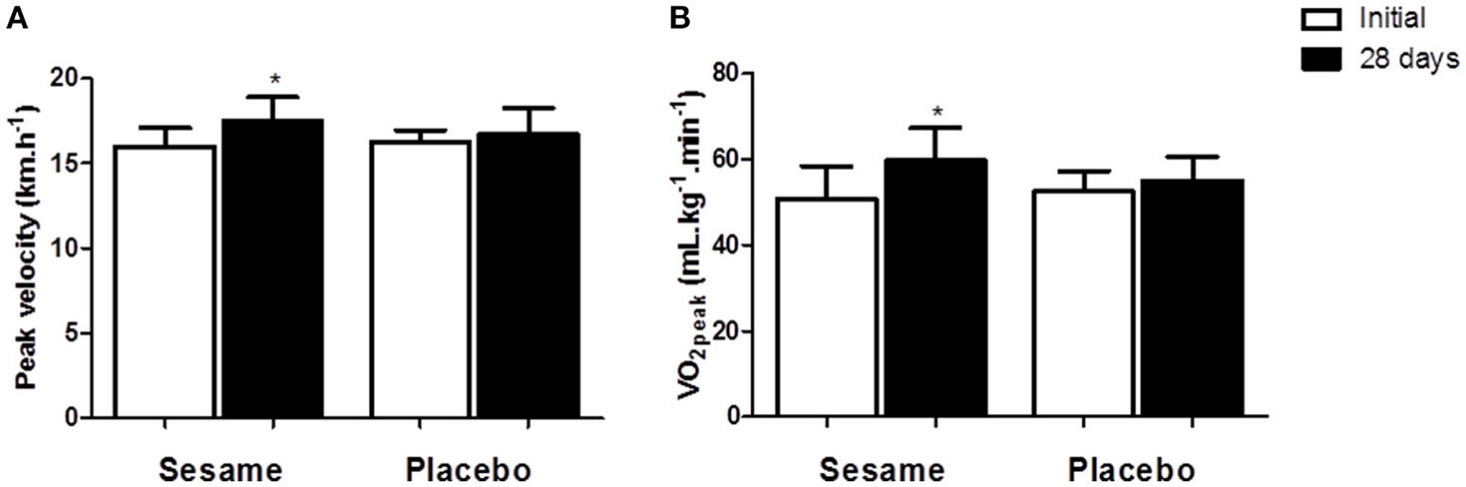
**Maximal aerobic capacity (A)** and peak aerobic capacity speed **(B)** before and after supplementation with sesame paste (*Sesamum indicum* L.) or placebo. Data are expressed as average ± standard deviation. ^*^Indicates intra-group differences between pre- and post-supplementation (two-way ANOVA with Tukey's test).

The intake of sesame paste caused a significant decrease in serum CK levels of ~19% in the experimental group relative to the initial values. At the end of the assessed period, serum CK in the experimental group was 5% lower than in the placebo group (*P* < 0.0001; Figure 1A). CK levels were higher in the placebo group at the end of the experiment (515.2 ± 15.81 U/L) than at the beginning (584.3 ± 10.48 U/L). Athletes who consumed the sesame paste showed a significant decrease in LDH enzyme at the end of the intervention period with *P* = 0.0184 (37% lower than baseline; Figure 1B). The placebo group showed no change in this variable, but the final values for the experimental group were only descriptively lower than the values found in the placebo group. Both groups showed a significant reduction in hs-CRP between the beginning and end of the study (experimental group *P* = 0.0006; placebo group *P* = 0.0020), with no differences between the groups at the end of the study (Figure 1C). MDA levels in the experimental group decreased significantly compared to baseline (*P* = 0.0144; Figure 2A). The placebo group also showed a significant reduction of MDA (P = 0.0091), but the reduction observed in the experimental group was greater (~55 vs. 21%), and supplemented athletes completed the study with values significantly lower than in the placebo group (0.7 ± 0.07 μmol/L for experimental group and 1.3 ± 0.04 μmol/L for placebo). SOD increased significantly after supplementation (~14%; *P* = 0.0022), while no change occurred in the placebo group (Figure 2B). The improvement in oxidative stress markers and antioxidant capacity observed in the experimental group was accompanied by significant increases in both serum vitamin A (25%) and vitamin E (65%). Vitamin A did not change in the placebo group, and vitamin E showed a significant increase in this group (~14%). However, this increase was much lower than that observed in the experimental group. Thus, the final vitamin A and E values were significantly higher in the experimental group than in the placebo group (*P* = 0.0009 and *P* = 0.0005 to vitamin A and E, respectively; Figures 2C,D).

**Figure 3:**
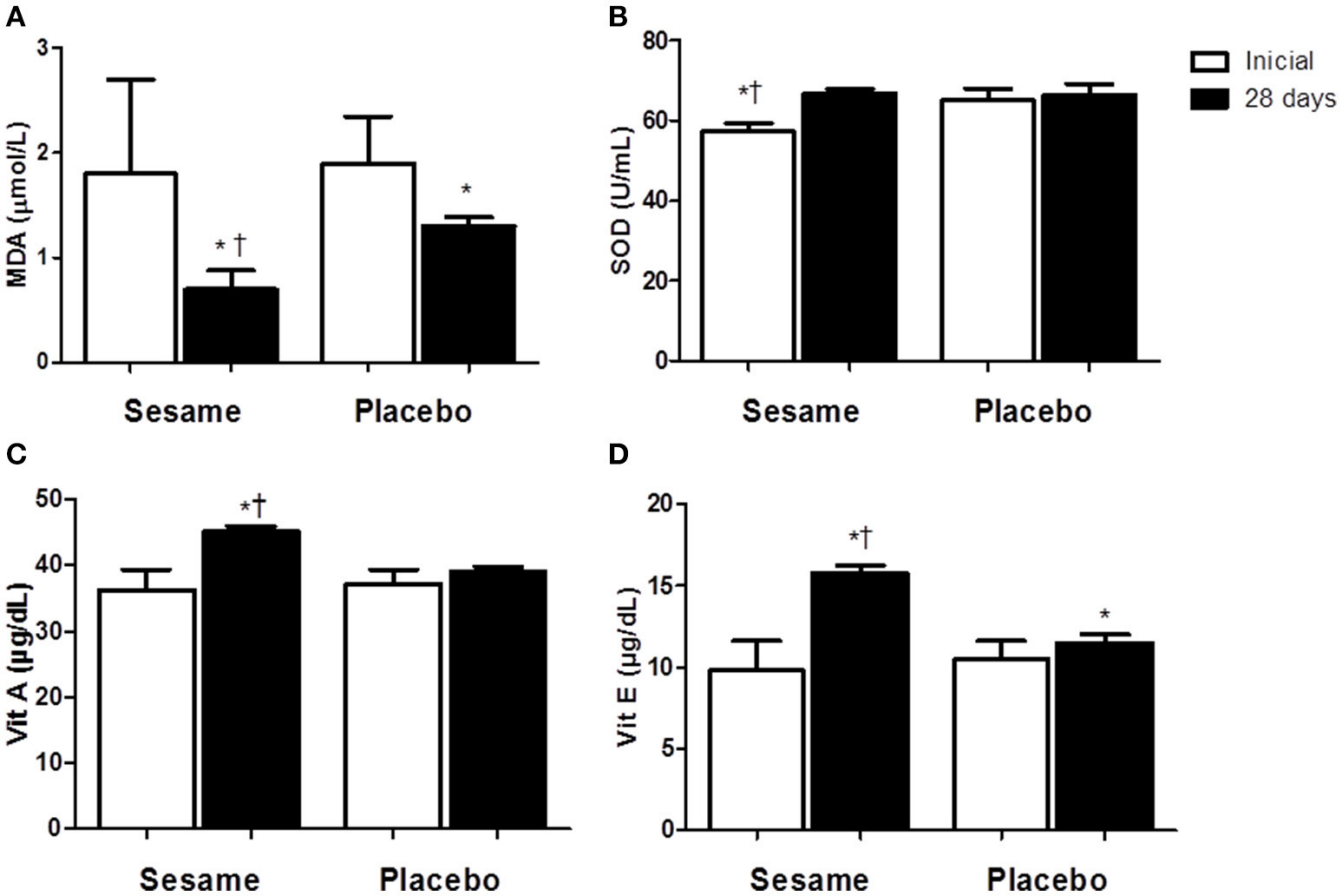
**Effect of sesame paste supplementation (***Sesamum indicum*** L.) on lipid peroxidation (A)** and enzymatic (SOD, **B**) and non-enzymatic antioxidants (vitamin A and E—**C,D**). Data are expressed as average ± standard deviation. ^*^Indicates intra-group differences between pre- and post-supplementation; ^†^Indicates difference between groups at the end of the study (Two-way ANOVA with Tukey's test). MDA, malondialdehyde; SOD, superoxide dismutase enzyme; Vit, vitamin.

The experimental group showed significant increases in aerobic capacity (~17%) and VO_2_ peak speed (~10%), as shown in Figures 3A,B. No change was observed in the placebo group. However, the final values for these two variables did not differ between groups.

## Discussion

Biochemical markers commonly used for diagnosis of physiological state (Meeusen et al., 2013) and laboratorial reference parameters indicated physiological imbalance at the beginning of the study for the status of CK and LDH baseline levels. The athletes were already in the final months of a season, which explains the wear found present at the start of this study. This wear is commonly observed at the end of the season in soccer players (Handziski et al., 2006; Silva et al., 2014).

The observed deficiency in intake of vitamins A and E is probably related to the deficient intake of foods considered good sources for these vitamins by Brazilian adolescents (Tureck et al., 2013). After 28-day supplementation with the sesame paste, these two antioxidant vitamins (A and E) increased in the serum of the athletes, supporting the reported nutritional benefits of sesame (Anilakumar et al., 2010). The increase of serum vitamin E after sesame seed supplementation has been reported in rats (Yamashita et al., 1995; Hanzawa et al., 2013). It has been suggested that sesame lignans regulate vitamin E metabolism by inhibiting tocopherol catabolism (Uchida et al., 2007; Hanzawa et al., 2013). However, other factors related to the effects of training on athlete's metabolism could have influenced the increase of vitamin E, once it was also observed in placebo group.

The antioxidant activity of sesame seeds has been attributed to a synergistic effect of vitamin E with lignans that act to detoxify the hydroxyl radicals, inducing a reduction in lipid peroxidation and, consequently, in MDA level (Namiki, 2007; Askari et al., 2012). At the present time, we do not have explanations to justify the reduction of MDA serum levels in placebo group at the end of the study period. However, considering that this reduction was accompanied by an increase in vitamin E, it could be related to a decrease in lipid peroxidation. The inversely proportional relation among vitamins A and E and lipid peroxidation is well-known. The modulation of oxidizing agents production by intake of antioxidant vitamins (e.g., vitamins A and E) was previously described (Peuchant et al., 1997).

The reduction of the inflammation marker hs-CRP could be related to the antioxidant activity because the oxidative stress directly associated with the inflammatory response (Haddad, 2002). Finally, ROS formation plays an important role in the etiology of muscle injury and the lipid peroxidation due to increased production of ROS can lead to the release of muscle constituents (Taghiyar et al., 2013). This set of interactions between inflammatory processes, oxidative stress, and muscle damage could partially explain the changes in markers of oxidative stress and muscle damage markers observed in athletes supplemented with sesame paste. Further studies with larger number of participants and additional markers could help to clarify these interactions.

Interestingly, the increase in maximal aerobic capacity occurred even without any aerobic-focused training; the athletes were in the final stage of the season, when training focuses on strength, speed and technical-tactical elements. In fact, reduced aerobic capacity or absence of gains (but no increase) is expected in the final half of a soccer season (Kalapotharakos et al., 2011). Previous study reported an improved aerobic capacity concomitant with reduction of oxidative stress markers in runners supplemented with grape juice (Toscano et al., 2015). However, here it is not yet clear by which mechanisms reducing oxidative stress or systemic inflammation contributes to better aerobic capacity.

## Conclusion

The results suggest that sesame consumption could be a nutritional strategy to improve the aerobic capacity of soccer players and to reduce important markers of oxidative stress and muscle damage.

## Ethics statement

This study was carried out in accordance with the recommendations of Resolution 196/96 of the Brazilian National Health Council (CNS) with written informed consent from all subjects. All subjects gave written informed consent in accordance with the Declaration of Helsinki. The protocol was approved by the Committee on Ethical Research Involving Humans Beings of the Federal University of Paraíba (João Pessoa, Brazil) under the Process Number 04308612.2.0000.5183.

## Author contributions

CB participated in elaboration, execution and writing of the study. CO fulfilled the analyzes of MDA and CK, and has participated in the implementation of dietary survey. NM helped to prepare the products and monitoring of training. YS, WC, and RC performed the physical-chemical and microbiological characteristics of the product. ACS offered all technical and scientific support about cultivating sesame that was used in the study. MM, ASS, PD, and VB participated in the elaboration, coordination or writing of the study.

### Conflict of interest statement

The authors declare that the research was conducted in the absence of any commercial or financial relationships that could be construed as a potential conflict of interest.
